# Supplementary material for: How did a duplicated gene copy evolve into a restorer-of-fertility gene in a plant? The case of Oma1
Source: R Soc Open Sci. 2019 Nov 6;6(11):190853. doi: 10.1098/rsos.190853 (PMC6894571; doi:10.1098/rsos.190853)
Supplement: Fig 3 for review [file rsos190853supp1.pptx]

## Slide 1
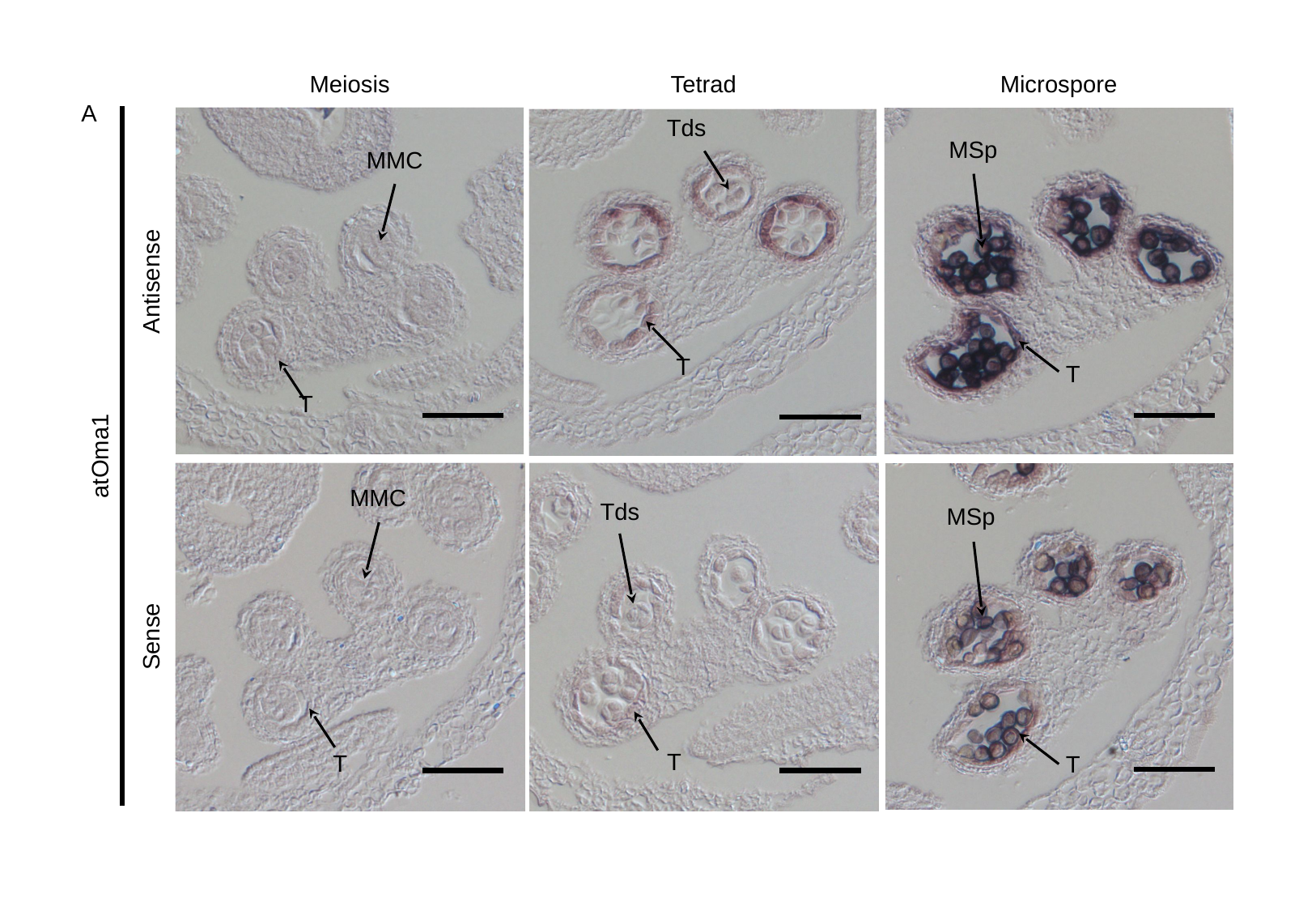

Meiosis
Tetrad
Microspore
A
MMC
T
Tds
T
MSp
T
Antisense
atOma1
MMC
T
Tds
T
MSp
T
Sense

## Slide 2
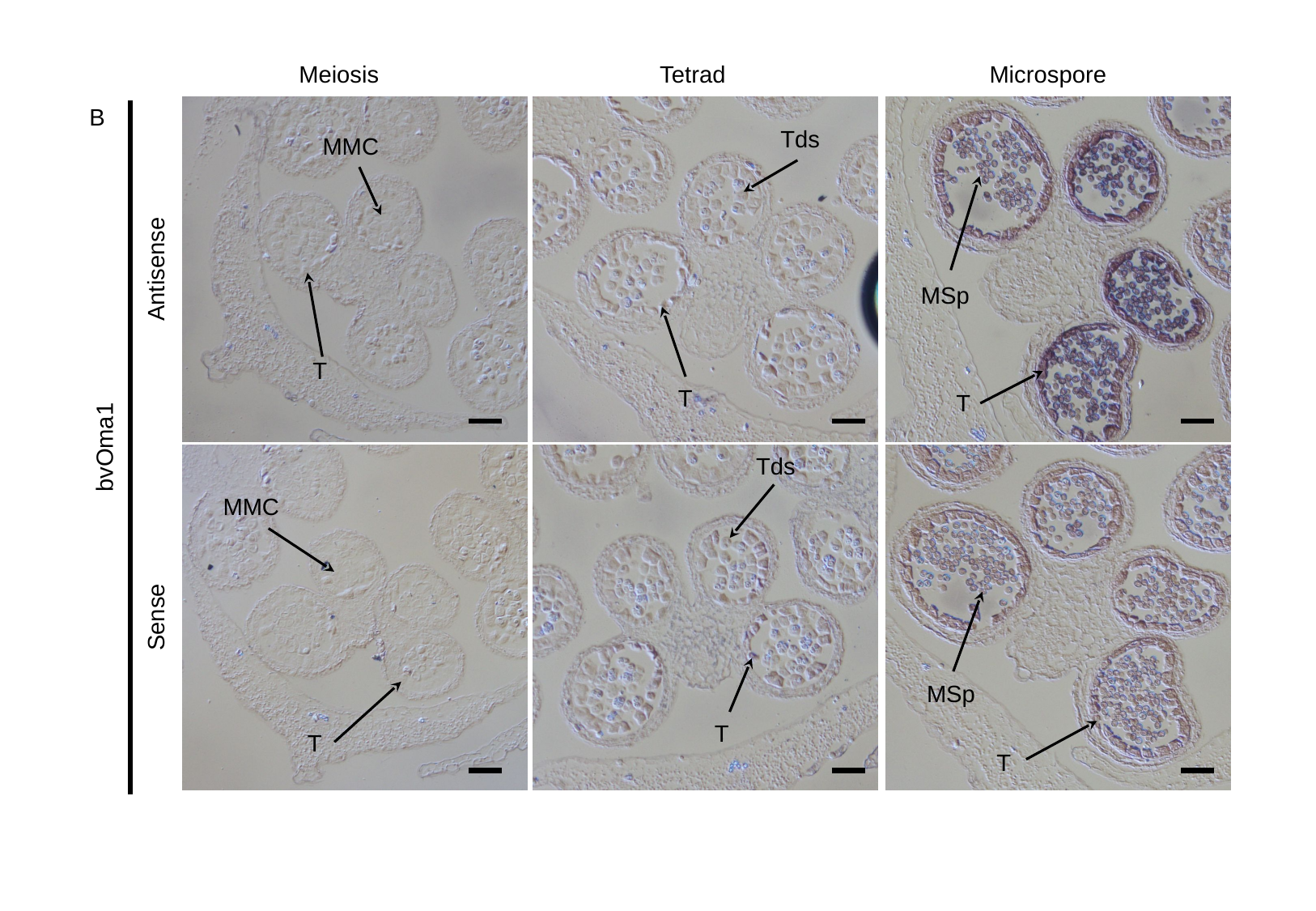

Meiosis
Tetrad
Microspore
B
MMC
T
Tds
T
MSp
T
Antisense
bvOma1
Tds
T
MMC
T
MSp
T
Sense

## Slide 3
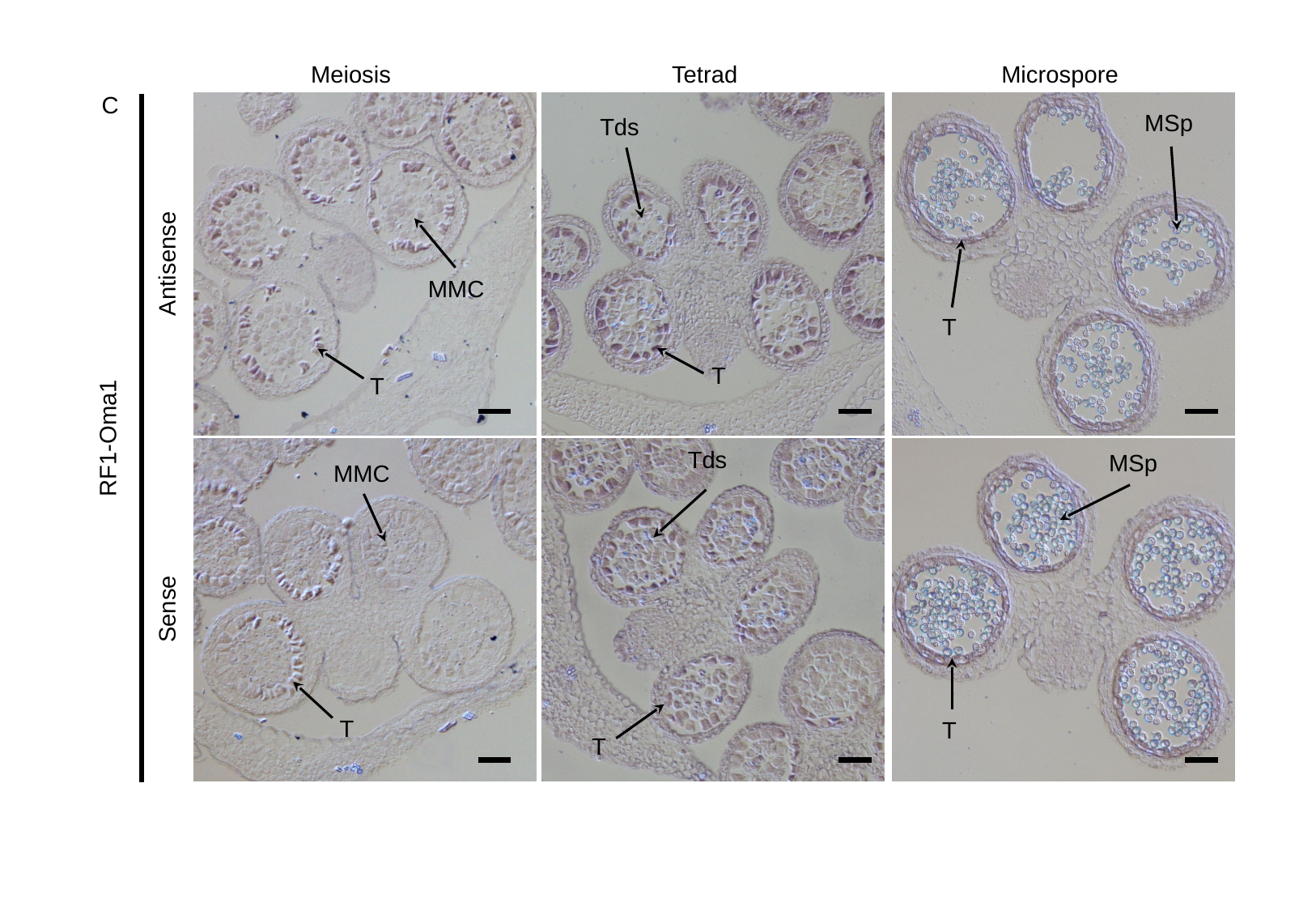

Meiosis
Tetrad
Microspore
C
MMC
T
Tds
T
MSp
T
Antisense
RF1-Oma1
MSp
T
MMC
T
Tds
T
Sense
