# Supplementary material for: How did a duplicated gene copy evolve into a restorer-of-fertility gene in a plant? The case of Oma1
Source: R Soc Open Sci. 2019 Nov 6;6(11):190853. doi: 10.1098/rsos.190853 (PMC6894571; doi:10.1098/rsos.190853)
Supplement: Fig S1 [file rsos190853supp2.pdf]

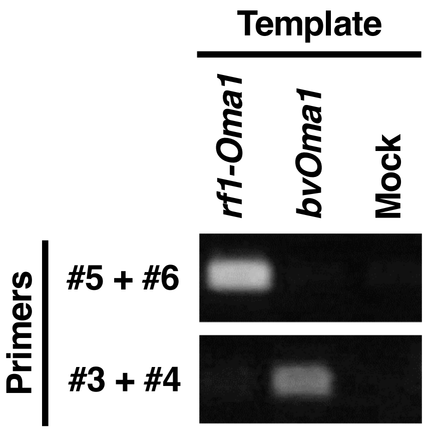

Fig. S1. Specificity of qRT-PCR primers.
Plasmid DNAs containing either *rf1-Oma1* (the binary vector used in [16]) or *bvOma1* (this study) were subjected to PCR using primers #5 and #6 or #3 and #4 with Blend Taq (Toyobo, Osaka, Japan). The PCR program was 30 cycles of 95˚C for 30 sec, 60.8˚C (the same temperature as used in the qRT-PCR assay) for 30 sec and 72˚C for 30 sec. The PCR products were electrophoresed in 2% agarose gels. The Mock sample contained all the components except the template DNA.
