# Supplementary material for: How did a duplicated gene copy evolve into a restorer-of-fertility gene in a plant? The case of Oma1
Source: R Soc Open Sci. 2019 Nov 6;6(11):190853. doi: 10.1098/rsos.190853 (PMC6894571; doi:10.1098/rsos.190853)
Supplement: Fig S2 [file rsos190853supp3.pdf]

```
                / Exon 1
bvOma1          ATGGCATGGT ACAGAAGATC AAGGTTTGTC TACAATGCTT ATAAAAGCTT 50
LOC104906603    ATGGCATGGT ACAGAAGATC AAGGTTTGTC TACAATGCTT ATAAAAGCTT 50

bvOma1          GAATTCCAAG TTATTATTGC CTAAAAGTCC AGTTCAATCT CCTATTCCAA 100
LOC104906603    GAATTCCAAG TTATTATTGC CTAAAAGTCC AGTTCCATCT CCTGTTCCAA 100

bvOma1          GATTTAATTC CAATTCATCT TCTTTGTTTT ACAATCAATT TAAGTCTTCT 150
LOC104906603    GAATTAATTC CAATTCATCT TCTTTGTTTT ACAATCAATT TAAGTCTTCT 150

bvOma1          ATAATTTCTG GGTCACCTTC AATTTCTTCA AAATTTGGGT ATTTGAATGG 200
LOC104906603    ATAATTTCTG GGTCACCTTC AATTTCTTCA AAATTTGGGT ATTTGAATGG 200

                Primer #1
bvOma1          GGTTAAACAG AATCAAAGTA GCTTGTTTTC TTGTGTTACT AGGAGAAATT 250
LOC104906603    GGTTAAACAG AATCAAAGTA GCTTGTTTTC TTGTGTTACT AGGAGAAATT 250

bvOma1          ACCATGTTGA TAGAAACCAA ATTTACCATT TTAAACCAAG AGGTTTTAAA 300
LOC104906603    ACCATGTCGA TACAAACCAA ATTTACCTTT TT-------- ---------- 282

bvOma1          TCTTGGTTTG AGAATCCTAG ACATATATTC ATCGCAGTAG TGATTGGTTC 350
LOC104906603    ---------- ---------- ---------- ---------- ---------- 282

bvOma1          TGGTGTTGTG ATCACTGTTT ATTTTGGCAA TTCAGAAGTT GTGCCCTATA 400
LOC104906603    ---------- ---------- ---------- ---------- ---------- 282

bvOma1          CAAAAAGGAA ACATCTTGTA CTTTTGTCAA GAACCCTAGA GAGGAGAATT 450
LOC104906603    ---------- ---------- ---------- -----CTAGA GAGGAGATAT 297

bvOma1          GGGGATTCTC AATTTGAGAA GATGAAGGAA GAGTTTAAGG GGAAAATATT 500
LOC104906603    GGGGAATTTC GATTTGAGAA GAGGAAGGAA GATTTTAAGG GGAAAATATT 347

                Primer #2
bvOma1          GCCTGCAATA CACCCTGATA GTGTGAGGGT TAGGTTGATA TCTAAAGACA 550
LOC104906603    GCCTGCAATA CACCCTGATA GTGTGAGGGT TAGGTTGATA TCTAAAGACA 397

bvOma1          TAATTGAGTC ATTAGAAAGA GGGATAAGCC ATGAAAGAGC ATGGAGTAGT 600
LOC104906603    TAATTGAGTC ATTAGGAAGA GGGATAAGCC ATGAAAGAGC ATGGAGTA-- 445

bvOma1          CCTGGATACG CCACCGAAAG CGTTAGCCAT CACGAGATCG ATGGGCATGA 650
LOC104906603    ---------- ---------- ---------- ---------- ---------- 445

bvOma1          AACTATGAAG GCATTAACTG AGGGGATGGA TGAGAAAGTG CCAGGGGATT 700
LOC104906603    -----CGAAG GCATTAACTG AGGGGATGGA TGAGAAAGTG CCAAGGGATT 490

bvOma1          GGCATAAGGA GGAGGAGGTT CTTGATGATA AGTGGGTTAA AGATAGTAGG 750
LOC104906603    GGCATAAGGA GGAGGAGGTT CTTGATGATA AGTGGGTTAA AGATAGTAGG 540
                                  Primer #3
bvOma1          AAGAAGGGGG AGAAACATGG GGCTAAGACT ACTACAAACC ATTTGGAGGG 800
LOC104906603    AAGAAGGGGG AGAAACATGG GGCTAAGACT ACTACAAACC ATTTGGAGGG 590

bvOma1          ATTGAATTGG GAAGTTCTGG TTGTGAACGA ACCAGTTGTA AATGCCTTTT 850
LOC104906603    CTTGAATTGG GAAGTTCTGG TTGTGAATGA ACCATTTGTA AATGCCTCTT 640
                                       Primer #4
bvOma1          GTTTACCAGG TGGGAAGATT GTTGTTTTCA CTGGATTGCT CAAGCATTTC 900
LOC104906603    ATTTTCCAGG TGGGAAGATT GTTGTTTTCA CTGGATTGCT CAAGCATTTA 690

                                                   Exon 1 /  Intron 1
bvOma1          AAATCAGATG CTGAATTGGC TACAATTATT GGACATGAGG TATACTATAG 950
LOC104906603    AAATCAGATG CTGAATTGGC TACAATTATT GGACATGAGG TTTATTATAG 740

bvOma1          CTTTGCTTTG CATAAATGCA ATCTTGGTTG ACATTAGTGA TTGATTGATT 1000
LOC104906603    CTTTGCTTTG CATAAATGCA ATCTTGGTTG ACATTAGGGT TTGATTGATT 790
```

```
bvOma1          ATATTATGTT ATATGAACAA TTAAGGAATT TTGTGAAATG AAACATTAGT 1050
LOC104906603    -----ATGTT ATATGAACAA TTAAGGAATT TTCTGAAATG AAACATTAGT 835

bvOma1          GATGATATTT TTAGGTGAGT TACATGATAA ATGTCATGTT CTGTGCTGGT 1100
LOC104906603    GATGATATTT TTAGATGAGT TACATGATAA ATGTCATGTT CTGTGCTGGT 885

                    Intron 1 / Exon 2
bvOma1          ATCTTTTTGT T--AGGTTGG ACATGCTGTG GCTCGACATT CTGCAGAACA 1148
LOC104906603    ATCTTTTTTT TTTAGGTTGG ACATGCTGTG GCTCGACATT CTGCAGAACG 935

bvOma1          AATTACAAAG AATATGTGGT TTGCAATCTT GCAACTGATC CTTTATCAAT 1198
LOC104906603    AATTACATGG ATTATGTCGT TTGCAAGCTT GCAACTGATC CTTC------ 979

bvOma1          TCATCGCGCC TGATTTTGCT AATGCAATGT ---------- ---------- 1228
LOC104906603    TCATCGCACT GGATTTTGCT TATGCAAGAT ATTTACAAAT ATCTTCATAT 1029

                                             Exon 2  / Intron 2
bvOma1          -CAAATCTTC TTTTAAGGCT TCCTTTTTCC CGAAAGTAAG TCTGTTACTC 1277
LOC104906603    GTAGGTGTTC TTTTAATTCT TTCTTTTGAC CGAAAGTAAG TCTGTTACTC 1079

bvOma1          CCAAAATGTT CTTTTTGATG ATTAACTTTG GCATTGGCTT TCTGGTTTAT 1327
LOC104906603    CCAAAATGTT CTTTTTGATG ATTAACTTTG GCATTGGCTT TCTGGTTTAT 1129

bvOma1          GGAGTTCCTT AGAATGTAAC GTGTCACAAT GTTAAAGTAA CT-GAGTTAA 1326
LOC104906603    GGAGTTCCTT AGAATGTAAG GTGTCACAAT GTTAAATTAA CTAGAGTTAA 1179

bvOma1          ATCTTCTGTT TCGTCTTGGT TAGAAGATGG TTAGATGATA TCACACTGAA 1376
LOC104906603    GTGTTCTGTT TCTTCTTGGT TAGAAGATGG TTAGATGATA TCACACTGAA 1229

bvOma1          TAAGAAATAC TGATACCATT TTTGCACAGT TTAAAGGCCA CGATATTGAG 1426
LOC104906603    TGAGAAATAC TGGTACCATT TTTGCACAGT TTAAAGGCCA CGATATTGAG 1279

bvOma1          GAACCTGAGC ATGAGAATGT TTAGCAAAAA TCACTAGACT GTTATGGTTT 1476
LOC104906603    GAAACTGAGC ATGAGAATGT TCAGCAAAAA TCACTAGACT GTTATGGTTT 1329

bvOma1          ATGTGGGAGT TGCTTATTGT CAAAATCGTT TCTGTAGTTT TTGTACCTTA 1526
LOC104906603    ATGTGGGAGT TGCTTATTGT CATAGTCGTT TCTGTAGTTT TTGTACCTTC 1379

bvOma1          TGTATCTTGT CCGGTAAAAA CTTTGTTATA TGAATTTCTT CTTAATCGAA 1576
LOC104906603    TGTATCTTGT TCGGGAGAAA CTTTGTTATA TGAATTTCTT CTTAATTGTA 1429

bvOma1          GGAAGAGAAG ATGGTTTAAG CCTTAAGAGT TATAGTTTTC TACTAACTGG 1626
LOC104906603    GGAAGAGAAG ATGGTTTAAG CCTTAAGGGT TATAGTTTCC TACTAACCGG 1479

bvOma1          TGCTTTTAGA TTTGAGTGGG -GAGAGAGCA GTAAGGAGGT GACTTGAGAG 1675
LOC104906603    TGCTTTTAGA TTTGAGTGGG AGGGAGAGCA GTAAGGAGGT GACTTGAGAG 1529

bvOma1          TATGATATGT AAAGACTAGA GACTAATTGC ATGGTCTGTT ---------C 1716
LOC104906603    TATGATATCT AAAGACTAGT GACTAATTGC ATGGTCTGTT GACTCTGTTC 1579

bvOma1          TACTCAGGGA AAGAGGGAAG GATTGGTAAG TTAACTGTTA CTTTTAATTG 1766
LOC104906603    TACTCAGGGA AAGAGGGAAG GATTGGTAAG TTAACTGTTA CTTTTAAATG 1629

bvOma1          GATGTTTGGT TCCACCATTG AGGGAACACT TATTAGTTAT TACTATGGGA 1816
LOC104906603    GATCTTTGGT TCCACCGTTG AGGGAACACT TATTAGTTAT TACTATGGGA 1679

bvOma1          TTAAGGAGGG TAACCGAATG TGTTCATAAC TTCATATTAT CATAGGGTTA 1866
LOC104906603    TTAAGGAGGG TAACCTAAGG TGTTCATAAC TTCATATTAT CATAGGGTTG 1729

bvOma1          AGGAGGTAAT AATAGGGTGG AAACAGTTGG CCTTTGGAAA TGGAAATCAT 1916
LOC104906603    AGGAGGTAAT AATAGGGTGG AAACAGTTGG CATCAGGAAA TGGAAACCAT 1779

bvOma1          TGCCCCACCC CTCATACCAA ACGTAGTAAC AGAATTTGTT TGTTTGTTTC 1966
LOC104906603    TACCCCACCC CTCATACCAA ACATA-TAAC GGAATTTGTA TGTTTGTTTC 1829
```

```
bvOma1          GGTTTCTAAC ACTTACTGAT ACTTAGCAAT GTTTTAATCG TAGAGTTCCA 2016
LOC104906603    GGTTTCTAAC GCTTACTAAT ACTTAGCAAT GTTTTAATCG TAGAGTTTCA 1879

bvOma1          GAATTTCCTA ACTGGATTGC AATGTGCGTT TATAAACTTC CATCCTATGT 2066
LOC104906603    TAATTTCCTA ACTGGATTGC AATGTGCGTT TATAAACTTC CAT--TATGT 1929

bvOma1          TACTTGGATG TTATGTCACT CAAACTCTTC ATATCACCTA AAGTACTCGT 2116
LOC104906603    TACATGGATG TTATGTCACT CAAACTCTTC ATATCACCTA AAGTACTCGT 1979

bvOma1          GCCAGATTCT TTTGAGATTT GATATCCTTT CCCTTAACTT TAAGCAGTAA 2166
LOC104906603    GCCAGATCCT TTTGAGATTT GATATCCTTT CCCTTAACTT TAAGCAGTAA 2029

bvOma1          TTGCCAATAT GATGTGATTA TTTTGTTTTT GAGTATGGAC ATTTTCTTTG 2216
LOC104906603    TTGCCAATAT GATGTGATTA ATTTTGTTTT GGGTATGGAC ATTTTCTTTT 2079

bvOma1          AACGTGGATC TAGTCGCATT CTCTGTGTGC TTCTATTCAT GCATTAGAAT 2266
LOC104906603    AACGTGGATC TAGTCGCATT CTCTGTGTGC TTCTATTCAT GCATTAGAAT 2129

bvOma1          AAAGAACATC ATGTCCTGGT CACTCTTTTC TGAAAGATTC TTGCCAATAT 2316
LOC104906603    AAAGA---TC TTGTCCTGGT CACTCTTTTC TGAAAGATTC TTGCCAATAT 2176

bvOma1          GACTGGTTTC TGGTGACCAT TGTTTACTAT GGGGATTTTG GTTGACCTGA 2366
LOC104906603    GACTAGTTTC TGGTGACCAT TGTTTACTAT GGGGATTTTG GTTTCCCTGA 2226

bvOma1          AAAGGGTCTC TCTTTAATTT ATTGCACGTA CCACTTGGAG GTGGCTCACT 2416
LOC104906603    AAAGTGTCTC TCTTTAATTT ATTGCACGTA CCACTTGGAG GTGGCTCACG 2276

bvOma1          GAATTCACTT TTGACTTATT GCTGAGGAAA TTGTCATCTC CTGTTTCATA 2466
LOC104906603    GAATCCACTT TTGACTTATA GCTGAGGAAA TTTTCATCTC CTGTTTCATA 2326

bvOma1          TCCCTCTCCG TAGATTGCTT TTTCTTTCTC CTTTGTATTT TAAAAATGGA 2516
LOC104906603    TCCCTCTCCG TAGATTGCTT TTCCTTTCTA CTCTGTATTT TAAAAATGGA 2376

bvOma1          AAATAGCTGG TGAAACATGA TGTACATGTG AGATGTGACA TTTGCAACAT 2566
LOC104906603    AAATAGCTGG TGAAACATGA TGTACAT-CG AGATGTGACA TTTGCAACAT 2425

bvOma1          AGGGTGTCAA TTAAAGAGTT AAAAAGTTCA TTGAGGCTAG AGCTCGTCCA 2616
LOC104906603    ATGGTGTCAA TTAAAGAGTT AAAAAGTTCA TTGAGGCTAG AGCTCGTTCC 2475

bvOma1          GTATGGCCAG CTGCTCGCTT GATCCAATTT GGACTAATTT ACTCCGGGTT 2666
LOC104906603    GAATGGCAAG CTGCTCGCTT GATCCAATTT GGACTGATTT ACTCCAGGTT 2525

bvOma1          TAAGTCGCCA TATGATTAGA TCTCCAATCC ACCTTTTCCA AATTGGTCCG 2716
LOC104906603    TAAGTTGCCA TATGATTAGA TCTCCAAACC ACCTTTTCCA AATTGGTTCG 2575

bvOma1          CCGCTTTTGG TGGTGTGGCC TGACCCAAAG TGTTATTTGA TCATTTTCAT 2766
LOC104906603    CCGCTTTTGG TGGTGTGGCC CGACCCAAAG TGTTATTTGA TCATTTTCAT 2625

bvOma1          CCTTCAACAT ATTTAACTTG TTTTCTATAG TTTTACCATT TAGTCATGTT 2816
LOC104906603    CCTTCAACAT ATTTAACTTG TTTTCTATAG TTTTACTATT TAGTCATGTT 2675

bvOma1          TGTGATGGGC TGATGGCACA AGTGAGCTCA CACATGCGAT GTCTGTATAC 2866
LOC104906603    AGTGATGGGC TGATGGCATA AGTGAGCTCG CTCATGCGAT GTCTGTATAG 2725

bvOma1          CTCAGCCCGG ATACCTAAGG CTATGATCCA GGTTCGCCTA ACCCCATATG 2916
LOC104906603    CTTAGCCTGG ATACCTAAGG CTATGATCCA GGCCCGCCTA ACCCCATATG 2775

bvOma1          TGCAACAAGC CTAAAACACA AGATGACAAG AAAACATTAC ACACTGACAC 2966
LOC104906603    TGCAAGAAGC CTAAAACACA AGATGACAAG AAAACATTAC ACACTGACAC 2825

bvOma1          TAACCTACAC CAAAACATTA TTCGTATCGG TGGACTTAGC ATGGGAATTT 3016
LOC104906603    TAACCTACAC CAAAACATTA TTCGTATCAG TGGACTTAGC ATGGGAATTT 2875

bvOma1          TTTCATATGG GAGCCGAAGC GGGAGAACTG TCAACCCATG ATAACATGCT 3066
LOC104906603    TCTCATATGG GAGCCGAAGC GGGAGAACTG TCAACCCATG ATAACATGCT 2925
```

```
bvOma1          CTTCTACCCT TAGATCTATC ATTCTGTGTT GCTGGTTTAT AAAAGCTTTT 3116
LOC104906603    CTTGTACCCT TAGATCTATC ATTCTATGTT GATGGTTTAT AAAAGCTTTT 2975

bvOma1          GCAAGAAAGC TGGTTTTGGT TCGATTTTTC TTTTGTTATG ACTTTATGTT 3166
LOC104906603    GCAAGAAAGC TGGTTTTGGT TT-ATTTTTC TTTTGTTATG ACTTTATGTT 3025

bvOma1          GTTTCAAAAT ACGAATTGTT TAAGCGTCCT TTTTTTCAA- TTTTGTATCG 3215
LOC104906603    GTTTCAAAAT ATGAATTGTT TAAGCGTCCT TTTTTTCAAG TTTTGTATCG 3075

                    Intron 2  / Exon 3
bvOma1          TTATTTGTGT AGAATGGAAA TAGAAGCAGA TTACATTGGA CTGCTTCTGA 3265
LOC104906603    TTATTTGTGT AGAGGGGAAA TAGAAGCAGA TTACATTGGA CTGCTTCTGA 3125

bvOma1          TGGCTTCTGC TGGATACGAC CCACGAATTG CACCTCAAGT ATATGAGAAG 3315
LOC104906603    TGGCTTCTGC TGGATACGAC CCACGAATTG CACCTCAAGT ATATGAGAAG 3175

bvOma1          CTGGGTAAGA TCTCTGGTGA ATCATCGTCG CTGACGGAAT ATCTCTCAAC 3365
LOC104906603    CTGGGTAAGA TCTCTGGTGA ATCATCATCG CTGAAGGAAT ATCTCTCAAC 3225

bvOma1          TCATCCATCG GGGAAAAAGC GTGCTCAGTT ATTAGCTCGA GCTCATATTA 3415
LOC104906603    TCATCCATCA GGGAAAAAGC GTGCTCAGTT ATTAGCTCGA GCTCATATTA 3275

bvOma1          TGCAAGAAGC AGTGGATATG TACCGTGAAA TTGTAGCAGG ACGCGCAATT 3465
LOC104906603    TGAAAGAAGC AGTGGATATA TACCGTGAAA TTGTAGCAGG ACACGCAATT 3325

bvOma1          GAAGGTTTTC TGTGA 3480
LOC104906603    GAAGGTTTTT TGTGA 3340
```

Fig. S2. Alignment of nucleotide sequences of *bvOma1* and *LOC104906603* of KWS2320. Exon/intron boundaries are shown by forward slashes. Dashes are incorporated for maximum matching. Nucleotide residues are numbered from the initiation codon. Positions of primers #1, #2, #3, and #4 are underlined.
