# Supplementary material for: How did a duplicated gene copy evolve into a restorer-of-fertility gene in a plant? The case of Oma1
Source: R Soc Open Sci. 2019 Nov 6;6(11):190853. doi: 10.1098/rsos.190853 (PMC6894571; doi:10.1098/rsos.190853)
Supplement: Fig S3 [file rsos190853supp4.pdf]

A

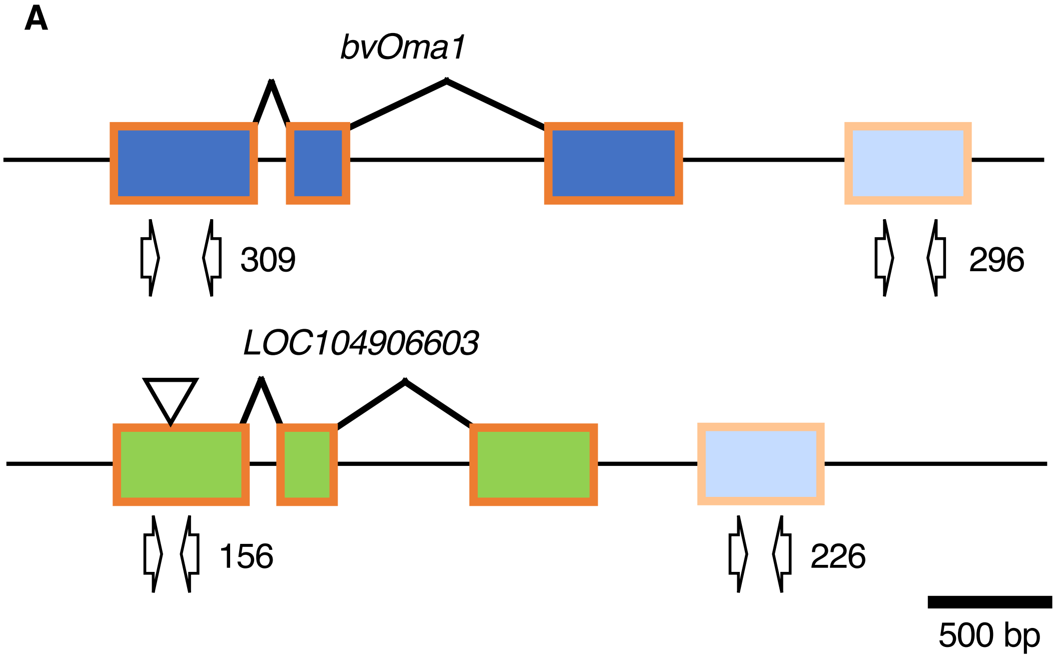

B

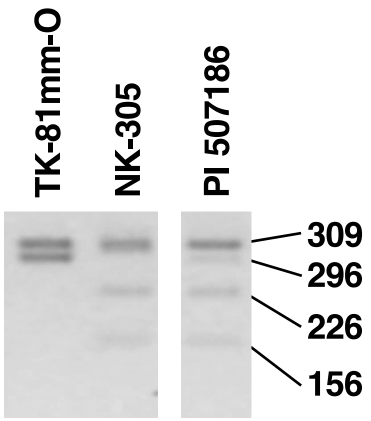

C

Line/ Accession	P/A of amplicon			
	309	296	226	156
TK-81mm-O	+	+	-	-
TA-33BB-O	+	-	-	-
NK-198	+	-	-	-
NK-219mm-O	+	-	-	-
NK-305	+	-	+	+
PI 507186	+	+	+	+

Fig. S3. PCR assay of beets indicated that *bvOma1* is preserved but *LOC104906603* is not among lines/accessions. A. Organizational comparison of *bvOma1* and *LOC104906603* in KWS2320. Deep-colored boxes and wedges indicate exons and introns, respectively. Pale-colored boxes indicate regions with homology to exon 1 of *bvOma1*. A triangle denotes the position of a deletion. Arrows indicate a pair of PCR primers (#1 and #2; see Fig. S2). The primer pair simultaneously targets four regions of the KWS2320 genome. The expected size of the PCR fragment is shown near the arrows. The scale bar is shown in the lower right corner. **B.** A representative electrophoretic pattern of PCR products using the primer pair shown in panel A. TK-81mm-O and NK-305 are sugar beet lines; PI 507186 is a wild beet (*Beta vulgaris* ssp. *maritima*) accession. Sizes of the amplicons are shown on the right. **C.** Genotype summary. Presence (P, +) or absence (A, -) of the four amplicons are tabulated. TA-33BB-O, NK-198 and NK-219mm-O are sugar beet lines.
