# Supplementary material for: How did a duplicated gene copy evolve into a restorer-of-fertility gene in a plant? The case of Oma1
Source: R Soc Open Sci. 2019 Nov 6;6(11):190853. doi: 10.1098/rsos.190853 (PMC6894571; doi:10.1098/rsos.190853)
Supplement: Fig S4 [file rsos190853supp5.pdf]

```
        / Exon 1
KWS2320 ATGGCATTTC ACAGAAATTC AAGGTTTGTC TACAATGCTC TAAAACCCAG 50
EL10    ATGGCATTTC ACAGAAATTC AAGGTTTGTC TACAATGCTC TAAAACCCAG 50
NK198   ATGGCATTTC ACAGAAATTC AAGGTTTCTC TACAATGCTC TAAAACCCAG 50

KWS2320 CTTCAATTCC AAGTTACTTA CTAAAACTTC ATCTCATTCC AATTCCTATT 100
EL10    CTTCAATTCC AAGTTACTTA CTAAAACTTC ATCTCATTCC AATTCCTATT 100
NK198   CTTCAATTCC AAGTTACTTA CTAAAACTTC ATCTCATTCC AATTCCTATT 100

KWS2320 CTTTGTTTTA CACTCAATTT AAGTATTCTA GGTTACATGG GTCTCCTTCA 150
EL10    CTTTGTTTTA CACTCAATTT AAGTTTTTTA GGTTACATGG GTCTCCTTCA 150
NK198   CTTTGTTTTA CACTCAATTT AAGTATTCTA GGTTACATGG GTCTCCTTCA 150

KWS2320 ATTTCTTCAA AATGTGGGTA CTTCAATGGG TTTAAACATA CTCAAAACAG 200
EL10    ATTTCTTCAA AATGTGGGTA CTTCAATAGG TTTAAACATA ATCAAAACAG 200
NK198   ATTTCTTCAA AATGTGGGTA CTTCAATAGG TTTAAACATA ATCAAAACAG 200

KWS2320 AATTATTTCT GGTGTTGCTA CTATCAGAAA TTCTCTTGAA GTTAAGAAAA 250
EL10    AATTATTTCT GGTGTTGCTA CTATCAGAAA TTCTCTTGAA GTTAAAAAAA 250
NK198   AATTATTTCT GGTGTTGCTA CTATCAGAAA TTCTCTTGAA GTTAAAAAAA 250

KWS2320 CTCAGAAATT CTATGGAAAA CAGCTAAAAA AAACTAATTC ATGGTGGGTG 300
EL10    CTCAGAATTT CTTTGGAAAA CAGCTAAAAA AAAAAAATTC ATGGTGGGGG 300
NK198   CTCAGAATTT CTTTGGAAAA CAGCTAAAAA AAACTAATTC ATGGTGGGGC 300

KWS2320 GACGGTATTT TTTTTGTGAT GACTATTTAT TATAGCATTT CAGAAGTTGT 350
EL10    GACGGTATTT TATTTGTGAT GACTATTTAT TATAGCATTT CAGAAGTTGT 350
NK198   GATGGTATTT TGTTTGTGAT GACTATTTAT TATAGCATTT CAGAAGTTGT 350

KWS2320 AC-------- CCTTTACAGA AAGGAAGCAT CTTGTGATTC CACTAACATC 392
EL10    AC-------- CCTTTACAGA AAGGAAGCAT CTTGTGATTC CGCTAACATC 392
NK198   ACACTTGTAC CCTTTACAGA AAGGAAGCAT CTTGTGATTC CACTAACATC 400

KWS2320 TCTTGAGATA AAAATTGGGG AATCTATGAA GAAGAAATTG TATGATGGGA 442
EL10    TCTTGAGATA AAAATTGGGG AATTTATGAA GAAGAAATTG TATGATGGTA 442
NK198   TCTTGAGATA AAAATTGGGG AATTTATGAA GAAGAAATTG TATGATGGTA 450

KWS2320 AAACATTGCA TGCTAGACAC CCTGCTAGTG TGAGGGCTAG AGTAGTATTT 492
EL10    AAATATTGCC TGCTACACAT CGTGCTAGTG TGAGGGCTAG AGTAGTATTT 492
NK198   AAATATTGCC TGCTACACAT CGTGCTAGTG TGAGGGCTAG AGTAGTATTT 500

KWS2320 GAACACATCA TTGTCTCTCT TGATCACAAG CTCATACATG AAGGGAATGG 542
EL10    GAACACATCA TTGTATCTCT TGATCACAAG CTCATACATG AAGGGCATGG 542
NK198   GAACACATCA TTGTATGTCT TGATCACAAG CTCATACATG AAGGGCATGG 550

KWS2320 ATCTAAGACA ACTACTAAGC ATTTGGAAGT TTTCGTTGTC GACGAACCTC 592
EL10    ATCTAAGACA ACTACTAAGC ATTTGAAAGT TTTCGTTGTC GACGAACCCC 592
NK198   ATCTAAGACA ACTACTAAGC ATTTGGAAGT TTTCGTTGTC GACGAACCTC 600

KWS2320 GGGTTTTTTC CTTTTGTTTT CCAGGTGGAA TGATTGCTGT TTCTACTGGG 642
EL10    GGGTTTTTTC CTTTTGTTTT CCAGGTGGAA TGATTGCTAT TTCTACTGGG 642
NK198   GGGTTTTTTC CTTTTGTTTT CCAGGTGGAA TGATTGTTGT TTCTACTGGG 650

KWS2320 TTGCTCAACT ATTTCCATTC AGATTCTGAA TTGGCTGCAA TTATTGGGAC 692
EL10    TTGCTCAACT ATTTCCATTC AGATTCTGAA TTGGCTGCAA TTATTGGGAC 692
NK198   TTGCTCAACT ATTTCCATTC AGATTCTGAA TTGGCTGCAA TTATTGGGAC 700

     Exon 1 / Intron 1
KWS2320 TCAGGTATAT AAGAGTATAC TTCATGATAG GGTTTTCTTG CAAACATACA 742
EL10    TCAGGTATAT AAGAGTATAC TTCATGATAG GTTTTTCTTG CAAACATACA 742
NK198   TCAGGTATAT AAGAGTATAC TTCATGATAG GTTTTTCTTG CAAACATACA 750

KWS2320 AAATTTTATT ATATCGAGAA TCATGTGGGA GCCTAATGGT TGG------- 785
EL10    AAGTTTTATT ATGTTGAGAA TCATGTGGGA GCCCAATGGT TGGATCTCCC 792
NK198   AAATTTTATT ATATCGAGAA TCATGTGGGA GCCTAATGGT TGG------- 793
```

```
KWS2320 ---------- ---------- ---------- ---------- ---------- 785
EL10    AACCTCATGG TCGTTAGTTC GACTCTCGTC AATCCCATAT TTGGGGTGTT 842
NK198   ---------- ---------- ---------- ---------- ---------- 793

KWS2320 ---------- ---------- ---------- ---------- ---------- 785
EL10    TGTGTGGATT TATGTTTCTG GATATAAATC TCCAGTTTCA GTCTTTGAAA 892
NK198   ---------- ---------- ---------- ---------- ---------- 793

KWS2320 ---------- ---------- ---------- ---------- ---------- 785
EL10    GTTTCCTGGT AGGTTGGGAT CCCGTTCTTA CCTTCAGCTC ATGAGGGTTG 942
NK198   ---------- ---------- ---------- ---------- ---------- 793

KWS2320 ---------- ---------- ---------- ---------- ---------- 785
EL10    AGTCTGCTTG GGCCAGCTAA TTACCAAATC AAAAAAAAAA TACAAAATTT 992
NK198   ---------- ---------- ---------- ---------- ---------- 793

KWS2320 ---------- ---------- ---------- ---TTGGAGC TCCCACCTCC 802
EL10    TATTATATTG AAAATCATGT TGGAGCCCAA TGGTTGGAGC TCCTATCTCC 1042
NK198   ---------- ---------- ---------- ---TTGGAGC TCCCACCTCC 810

KWS2320 CAACCTCATG GTCG------ ---------- ---------- --------TT 818
EL10    CAACCTCATG GTCGAGAGTT CGACTCTCAT CAACCCAAAA CGGGGTGTTT 1092
NK198   CAACCTCATG GTCG------ ---------- ---------- --------TT 826

KWS2320 GTGTGGATTT ATGCTTTTGG ATATAAATCT CGAGTTTTCA GTCT--GAAA 867
EL10    GTGTGGATTT ATGCTTCTGG ATATAAATCC CTAGTTTTCG GTCTCTGAAC 1142
NK198   GTGTGGATTT ATGCTTTTGG ATATAAATCT CGAGTTTTCA GTCT--GAAA 874

KWS2320 GTTTCCTTAT GGGTTGAGAT CCCCTTCTTA GCCCACCAGC TCATGAGGGC 917
EL10    GTTTCATTAT GGGTTGGGAT CCCCTTCTTA -CCTCCCAGC TAATGAGGAT 1191
NK198   GTTTCCTTAT GGGTTGGGAT CCCCTTCTTA GCCCACCAGC TCATGAGGGC 924

KWS2320 TGAGCTTGCT TGGGCTAGCT ATTTACCAAA TCAAAAAAAA GAA---TACA 964
EL10    TTAGCCTGCT TGGGCCAGCT AATTACCAAA TAAAAAAA-- ------TACA 1233
NK198   TGCGCTTGCT TGGGCTAGCT AATTTCCAAA TCGAAAAAAA AAAATATACA 974

KWS2320 AAATTTTATT ATATGAATAT TAGTTTAGTG ATGAAATTTT TGGTTGCTAT 1014
EL10    AAACTTTATT ATATGAATAT TAGTTTAGTG ATGAAATTTT TGGTTGCTAT 1283
NK198   AAATTTTATT ATATGGATAT TAGTTTAGTG ATGAAATTTT TGGTTGCTAT 1024

                                        Intron 1 / Exon 2
KWS2320 CATGATAATG TGATGTTATA TGGTGGTATG TTCTTGATAG GTTGCGGATG 1064
EL10    CATGATAATG TGATGTTATA TGGTGGTATG TTCTTGATAG GTTGCGGATG 1333
NK198   CATGATAATG TGATGTTATA TGGTGGTATG TTCTTGATAG GTTGCGAATG 1074

KWS2320 CTGTGGCTCG GCCTTTTGCA GAATTCTTTC CAAAGTATAT GTTGGCTATG 1114
EL10    CTGTGGCTCG GCCTTGTGCA GAACTCTTTC CAAAGTATAT GTTGGCTATG 1383
NK198   CTGTGGCTCG GCCTTTTGCA GAATTCTTTC CAAAGTATAT GTTGGCTATG 1124

                                 Exon 2 / Intron 2
KWS2320 TTTGTCATTT CGATTATCAA TCCCAGTGCC AGGTCTTATG AACATATCTG 1164
EL10    TTTGTCATTT CGATTATCAA TCCCAGTGCC AGGTCTTATG AACATATCTG 1433
NK198   TTTGTCATTT CGATTATCAA TCCCAGTGCC AGGTCTTATG AACATATCTG 1174

KWS2320 CCCCCTTTTT TTTTTCTTAG AGATTATTGC AAATATTGCT AGTTTCTAGT 1214
EL10    CCCC--TTTT TTTTTCTTAG AGATTATTGC AAATATTGCT AGTTTCTAGT 1483
NK198   CCCCCTTTTT TTTTTCTTAG AGATTATTGC AAATATTGCT AGTTTCTAGT 1224

KWS2320 GATCTTGGAT TACTTATTTT GTGGACTGTG GTTAACCTGA AATTTA---- 1260
EL10    GATCTTGGAT TACTTATTTT GTGGACTGTG GTTAACCTGA AATTCAAACA 1533
NK198   GATCTTGGAT TACTTATTTT GTGGACTGTG GTTAACCTGA AATTCA---- 1270

KWS2320 TTCTTGTCAA GATCCTCCTT GGAAATTGGA AATGACGCAC TCAATTCTCC 1310
EL10    TTCTTGTCAA AATC------ --AAATTGGA AATGACTCAC TCAATTCTCC 1575
NK198   TTCTTGTCAA GATCCTCCTT GGAAATTGGA AATGACGCAC TCAATTCTCC 1320
```

```
KWS2320 AGACTTCTCT GCTACTGATT TGCAAATTTC ATATCCTTCT ATGATATTCT 1360
EL10    AGACTTCTCT GCTACTGATT TGCAAATTTC ATGTCCTTCT ATGATATTCT 1625
NK198   AGACTTCTCT GCTACTGATT TGCAAATTTC ATATCCTTCT ATGATATTCT 1370

KWS2320 ATGCTTCTTT TTAGTCATTT AGTTGCAATA CTTTGGCACT TGGCAATATG 1410
EL10    ATGCTTCTTT TTAGTCATTT AGTTGCAATA CTTTGGCACT TGGCAATATA 1675
NK198   ATGCTTCTTT TTAGTCATTT AGTTGCAATA CTTTGGCACT TGGCAATATG 1420

KWS2320 CAACTTTGAG CATTAGTGTT GCTAGTCGAA GTTAGTATAT GAATAGTATG 1460
EL10    CAACTTTGAG CATTAGTGTT GCTAGTCGAA GTTAGTATAT GAATAGTATG 1725
NK198   CAACTTTGAG CATTAGTGTT GCTAGTCGAA GTTAGTATAT GAATAGTATG 1470

KWS2320 AAGGTCAAAA TATTGCAACT AATAAAAACA GAGAAGCTAC ATGGAAATTG 1510
EL10    AAGGTCAAAA TATTGCAACT AATAAAAACA GAGAAGCTAC ATGGAAATTG 1775
NK198   AAGGTCAAAA TATTGCAACT AATAAAAACA GAGAAGCTAC ATGGAAATTG 1520

KWS2320 AGTAGTGAAA CATGTTATAT GTGCATGTAA GTTTTGTGAC ATAGGGTGTC 1560
EL10    AGTAGTGAAA CATGTTATAT GTGCATGTAA GTTTTGTGAC ATAGGGTGTC 1825
NK198   AGTAGTGAAA CATGTTATAT GTGCATGTAA GTTTTGTGAC ATAGGGTGTC 1570

       Intron 2 / Exon 3
KWS2320 AATTTACAGA ATTGTGAAGA TTATTCAGGC TAGAGCTTGT AAACTACGGC 1610
EL10    GATTTACAGA ATTGTGAAGA TTATTCAGGC TAGAGCTTGT AAACTACGGC 1875
NK198   AATTTACAGA ATTGTGAAGA TTATTCAGGC TAGAGCTTGT AAACTACGGC 1620

KWS2320 CATTCACCGC TGGCTTGATC AAATCTGGAC TGAATTTTAC CGGGCTTCTT 1660
EL10    CATTCACCGC TGGCTTGATC AAATCTGGAC TGAATTTTAC CGGGCTT--- 1922
NK198   CATTCACCGC TGGCTTGATC AAATCTGGAC TGAATTTTAC CGGGCTT--- 1667

KWS2320 CTGCTGTGTT TTGCACCATT GGATTGTTAT TTTCGTTGGA GGAAGATGGA 1710
EL10    CTGCTGTGTT TTGCACCATT GGATTGTTAT TTTCTTTGGA GGAAGATGGA 1972
NK198   CTGCTGTGTT TTGCACCATT GGATTGTTAT TTTCTTTGGA GGAAGATGGA 1717

KWS2320 AGCAGATTAC ATTGGCCTAC AGTTGATGTC TTCTGCTGGA TACGACCCAC 1760
EL10    AGCAGATTAC ATTGGCCTGC AGTTGATGTC TTCTGCTGGA TACGACCCAC 2022
NK198   AGCAGATTAC ATTGGCCTGC AGTTGATGTC TTCTGCTGGA TACGACCCAC 1767

KWS2320 GAGTTGCACC TCAAGCATAT CAGAAGCTGA GAAGGCAAAC TATAGCTTAG 1810
EL10    GAGTTGCACC TCAAGCATAT CAGAAGCTGA GAAGGCAAAC TATAGCTTAG 2072
NK198   GAGTTGCACC TCAAGCATAT CAGAAGCTGA GAAGGCAAAC TATAGCTTAG 1817
```

Fig. S4. Alignment of nucleotide sequences of *LOC104888056* from KWS2320, EL10 and NK-198. Exon/intron boundaries are shown by forward slashes. Dashes are incorporated for maximum matching. Nucleotide residues are numbered from the initiation codon. An insertion of eight nucleotides that leads to a frame shift mutation is underlined. The resulting premature stop codon is highlighted in yellow.
