# Supplementary material for: How did a duplicated gene copy evolve into a restorer-of-fertility gene in a plant? The case of Oma1
Source: R Soc Open Sci. 2019 Nov 6;6(11):190853. doi: 10.1098/rsos.190853 (PMC6894571; doi:10.1098/rsos.190853)
Supplement: Fig S5 [file rsos190853supp6.pdf]

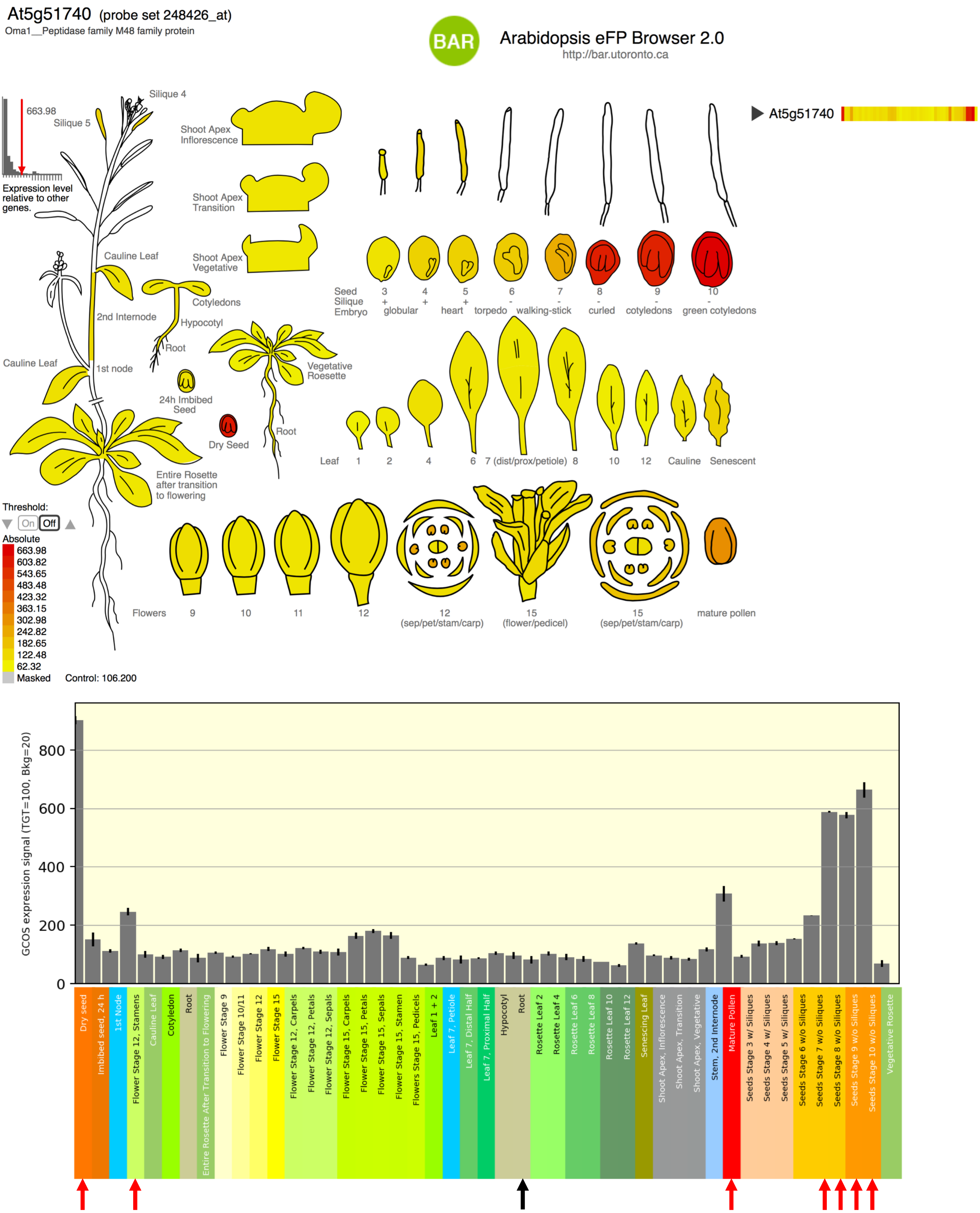

Fig. S5. Expression of *atOma1*. The microarray-based expression pattern of *atOma1* was retrieved from Arabidopsis eFPBrowser 2.0. In the lower panel, red arrows indicate the organs having expression values more than twice that of root (black arrow) .
