# Supplementary material for: How did a duplicated gene copy evolve into a restorer-of-fertility gene in a plant? The case of Oma1
Source: R Soc Open Sci. 2019 Nov 6;6(11):190853. doi: 10.1098/rsos.190853 (PMC6894571; doi:10.1098/rsos.190853)
Supplement: Fig S6 [file rsos190853supp7.pdf]

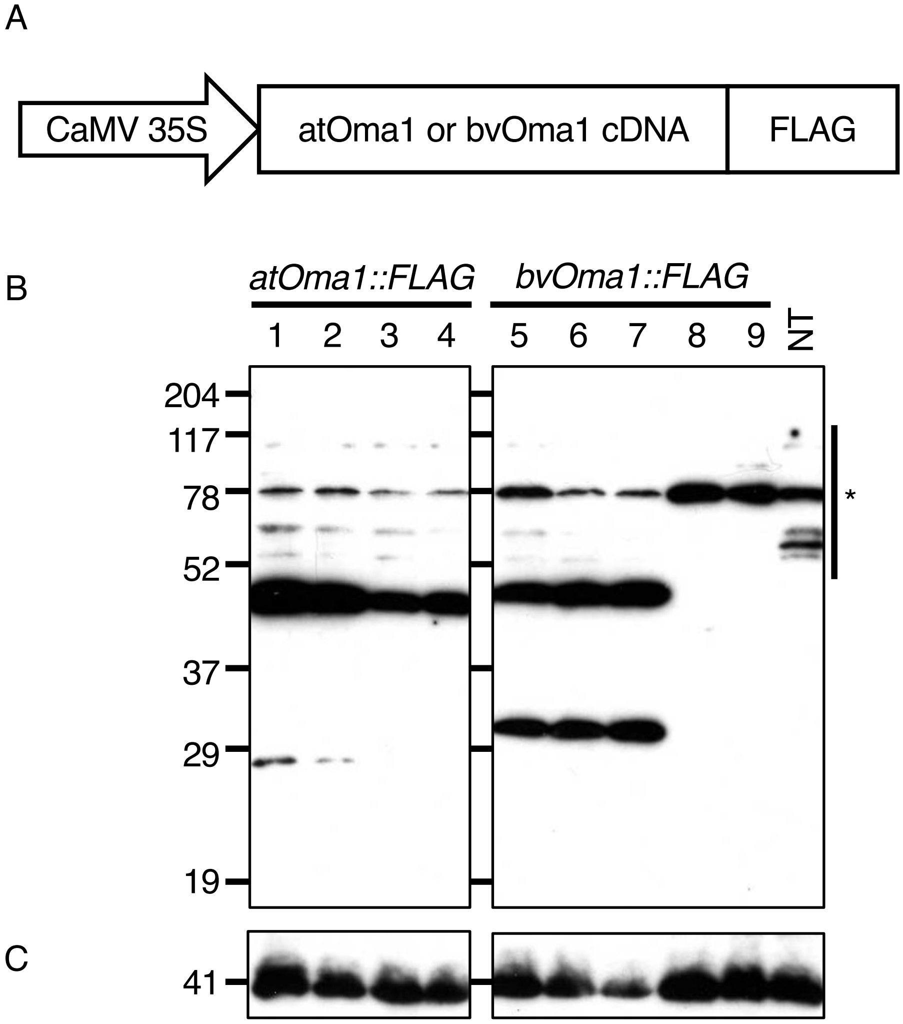

Fig. S6. Establishment of transgenic sugar beet suspension cells expressing *atOma1* or *bvOma1*. A. Organization of the transgene. The arrow indicates the 35S promoter sequence derived from cauliflower mosaic virus. Boxes show the coding regions of *atOma1* (or *bvOma1*) fused with a FLAG (DDDDK) tag. **B.** Protein gel blot analysis of sugar beet suspension cells probed with anti-FLAG. Protein samples were subjected to 12% SDS-polyacrylamide gel electrophoresis. Lanes 1 to 4 are cell lines expressing the CaMV35S-*atOma1*::FLAG transgene. Lanes 5 to 9 are those expressing the CaMV35S-*bvOma1*::FLAG transgene. NT denotes non-transgenic suspension cells (control). An asterisk denotes non-specific signal bands. No detectable transgene expression was seen from cell lines in lanes 8 and 9. Size markers are shown on the left (kDa). **C.** The same samples as panel B were probed with anti-COXI.
