# Supplementary material for: How did a duplicated gene copy evolve into a restorer-of-fertility gene in a plant? The case of Oma1
Source: R Soc Open Sci. 2019 Nov 6;6(11):190853. doi: 10.1098/rsos.190853 (PMC6894571; doi:10.1098/rsos.190853)
Supplement: Table S1 [file rsos190853supp8.pdf]

Table S1 Nucleotide sequences of primers used in this study

ID	Nucleotide sequence
#1	5'-GGTTAAACAGAATCAAAGTAGCTTG-3'
#2	5'-ATTGCAGGCAATATTTTCCCC-3'
#3	5'-ATGGGGCTAAGACTACTACAAACCA-3'
#4	5'-GAAAACAACAATCTTCCCACCTGG-3'
#5	5'-GGAAGAAGCATAGTGGGGCT-3'
#6	5'-CACAGCATGCCCAACCTGAT-3'
#7	5'-AGACCTTCAATGTGCCTGCT-3'
#8	5'-ACGACCAGCAAGATCCAAAC-3'
#9	5'-TGAGGCTGGTATCTCCAAGG-3'
#10	5'-TTGAGTACTTGGGGGTGGTG-3'
#11	5'-ACGCGTCGACCCATTGATCATCAAGAACCTGAT-3'
#12	5'-GCTCTAGACGATTCCCTGCGACGCAA-3'
#13	5'-GCTCTAGAATGGCATGGTACAGAAGATCAA-3'
#14	5'-ACGCGTCGACTTGTAGCCAATTCAGCATCTGATT-3'
#15	5'-AGCTTGCAAAGCCACTGGGCGA-3'
#16	5'-GGAACCAAATTAGATTGAATTAACAAGTGG-3'
#17	5'-GGGGACAAGTTTGTACAAAAAAGCAGGCTATGGCATGGTACAGAAGATCAAGG-3'
#18	5'-GGGGACCACTTTGTACAAGAAAGCTGGGTTCACAGAAAACCTTCAATTGCGCG-3'
#19	5'-GGGGACAAGTTTGTACAAAAAAGCAGGCTATGTCATGGTACAGAAGAACAAAAC-3'
#20	5'-GGGGACCACTTTGTACAAGAAAGCTGGGTCTAAAGAAAGCCTTCAACGCCAG-3'
#21	5'-TTTTCTGGATTATAAGGATGATGATGATAAGTGAACCCAGCTTTCTTGT-3'
#22	5'-GGGTTCACTTATCATCATCATCCTTATAATCCAGAAAACCTTCAATTGC-3'
#23	5'-CTTTCTTGATTATAAGGATGATGATGATAAGTAGACCCAGCTTTCTTGT-3'
#24	5'-GGGTCTACTTATCATCATCATCCTTATAATCAAGAAAGCCTTCAACGCC-3'
