# Supplementary material for: How did a duplicated gene copy evolve into a restorer-of-fertility gene in a plant? The case of Oma1
Source: R Soc Open Sci. 2019 Nov 6;6(11):190853. doi: 10.1098/rsos.190853 (PMC6894571; doi:10.1098/rsos.190853)
Supplement: Table S2 [file rsos190853supp9.pdf]

Table S2 Locations of *Oma1* -homologues on sugar beet chromosome 3 and scaffolds/contigs deposited in public databases.

<table>
<tr><th>Sugar beet line</th><th colspan="2">KWS2320</th><th colspan="2">EL10</th><th colspan="2">NK-198</th></tr>
<tr><th>Gene</th><th>Position</th><th>Source[1]</th><th>Position</th><th>Source[1]</th><th>Position</th><th>Source[1]</th></tr>
<tr><td rowspan="6">RF1-Oma1</td><td rowspan="6">2,397,803..2,400,302 complement</td><td rowspan="7">NC_025814.2
(Chr. 3)</td><td>2,453,935..2,455,800 complement</td><td rowspan="9">CM009440.1
(Chr. 3)</td><td>22,153..24,018 complement</td><td rowspan="2">AB646133.1
(Chr. 3)</td></tr>
<tr><td>2,460,941..2,462,823 complement</td><td>29,159..31,041 complement</td></tr>
<tr><td>2,471,512..2,473,394 complement</td><td rowspan="2">13,577..15,436 complement</td><td rowspan="5">AB646135.2
(Chr. 3)</td></tr>
<tr><td>2,485,364..2,487,246 complement</td></tr>
<tr><td>2,499,220..2,501,079 complement</td><td rowspan="2">21,533..23,398 complement</td></tr>
<tr><td>2,507,177..2,509,036 complement</td></tr>
<tr><td>LOC104888056</td><td>2,444,473..2,446,764</td><td>2,519,891..2,521,960</td><td>64,077..65,893</td></tr>
<tr><td>LOC104906603</td><td>301,862..306,206</td><td rowspan="2">NW_017567367.1
(Chr. 3)</td><td>-</td><td>Unknown</td><td rowspan="2"></td></tr>
<tr><td>bvOma1</td><td>327,302..331,422</td><td>10,738,367..10,741,893</td><td>Unknown</td></tr>
</table>

[1]Assigned chromosome is in parentheses.
