# Supplementary material for: How did a duplicated gene copy evolve into a restorer-of-fertility gene in a plant? The case of Oma1
Source: R Soc Open Sci. 2019 Nov 6;6(11):190853. doi: 10.1098/rsos.190853 (PMC6894571; doi:10.1098/rsos.190853)
Supplement: Table S3 [file rsos190853supp10.pdf]

Table S3 Quantitative Reverse Transcription-PCR analysis of *bvOma1* and *rf1-Oma1* in various organs

Tissue/Organ	Reference gene/Target gene			
	Actin		*ef1α*	
	bvOma1	*RF1-Oma1*	*bvOma1*	*RF1-Oma1*
Root	1.00±0.09[a]	1.00±0.91	1.00±0.12	1.00±0.95
Leaf	1.25±0.21	UD[b]	1.45±0.33	UD
Peduncle	1.08±0.08	UD	1.42±0.07	UD
Flower bud	1.19±0.90	115.90±64.61	1.38±1.09	123.40±75.93
Flower budΔanther	1.00±0.18	19.43±21.28	0.96±0.17	17.08±15.29
Immature anther	3.25±1.57	473.08±9.04	3.40±1.58	487.03±15.37

[a]Mean±SD, n=3; [b]Under detectable level.
